# A New Approach to Determine the Total Airborne N Input into the Soil/Plant System Using N Isotope Dilution (ITNI): Results for Agricultural Areas in Central Germany

**DOI:** 10.1100/tsw.2001.94

**Published:** 2001-10-03

**Authors:** Rolf W. B. Russow, Frank Bahme, Heinz-Ulrich Neue

**Affiliations:** UFZ Centre for Environmental REsearch Leipzig-Halle, Department of Soil Sciences, Halle, Germany

**Keywords:** airborne nitrogen, atmospheric N deposition, Central Germany, critical load, 15N dilution, N fertilization, soil/plant system

## Abstract

The atmospheric deposition of nitrogen (N) in the environment is of great concern due to its impact on natural ecosystems including affecting vegetation, reducing biodiversity, increasing tree growth in forests, and the eutrophication of aquatic systems. Taking into account the average annual N emission into the atmosphere in Germany of about 2 million t N (ammonia/ammonium, NO_x_), and assuming homogeneous distribution throughout Germany, an average N deposition of 45 kg/ha x year can be calculated. Such high atmospheric N deposition could be confirmed by N balances from long-term field experiments in Central Germany (e.g., the Static Fertilization Experiment in Bad Lauchstädt). By contrast, estimates by standard methods indicate a deposition of only about 30 kg N/ha x year. This is because the standard methods are using wet-only or bulk collectors, which fail to take into account gaseous deposition and the direct uptake of atmospheric N by aerial plant parts. Therefore, a new system was developed using N isotope dilution methodology to measure the actual total atmospheric N input into a soil/plant system (Integrated Total Nitrogen Input, ITNI). A soil/plant system is labeled with [N]ammonium-[N]nitrate and the total input of airborne N is calculated from the dilution of this tracer by N from the atmosphere. An average annual deposition of 64 ± 11 kg/ha x year from 1994–2000 was measured with the ITNI system at the Bad Lauchst?dt research farm in the dry belt of Central Germany. Measurements in 1999/2000 at three other sites in Central Germany produced deposition rates of about 60 kg/ha x year. These data clearly show that the total atmospheric N deposition into the soil/plant system determined by the newly developed ITNI system significantly exceeds that obtained from standard wet-only and bulk collectors. The higher atmospheric N depositions found closely match those postulated from the N balances of long-term agricultural field experiments.

## INTRODUCTION

The deposition of nitrogen (N) from the atmosphere on the landscape is a serious issue due to its impact on natural ecosystems (including altering vegetation, the loss of biodiversity, increasing tree growth in forests, and the eutrophication of aquatic systems[1]). Although this atmospheric N input represents free fertilizer for agriculture, it must be taken into account by N fertilizer recommendations designed to reduce the N surplus in agriculture, which is currently 100 kg N/ha × year or more in Germany[2,3]. This entails calculating atmospheric N deposition. Unfortunately, little is known about the magnitude or the spatiotemporal distribution of total N deposition.

The average annual N emission into the atmosphere in Germany amounts to about 2 million t N (ammonia/ammonium, $NO_x$). Assuming homogeneous distribution throughout the country, average N deposition of 45 kg/ha × year can be calculated[4]. N balances from long-term field experiments in Central Germany (such as the Static Fertilization Experiment in Bad Lauchstädt[5])

have confirmed such high atmospheric N deposition. However, current estimates using state-of-the-art methods such as wet-only and bulk collectors do not take into consideration the gaseous deposition and direct uptake of atmospheric N by aerial plant parts. Accordingly, these methods underestimate the actual atmospheric N input, which is the reason why they only indicate an average deposition of 30–35 kg N/ha × year for Germany. There are also different methods to measure and calculate the gaseous N deposition[6,7,8,9,10] but these methods are rather uncertain and the measuring processes need complicated equipment. Therefore, a new device using the $^{15}N$ isotope dilution methodology was developed to measure the actual total atmospheric N input (integrated total nitrogen input [ITNI]) into a soil/plant system. Total atmospheric N input means the sum of wet, dry (particulate matter), and gaseous deposition, including the direct N uptake by above-ground plant parts.

## EXPERIMENTAL METHODS

### The ITNI System

The ITNI measuring system is based on the $^{15}N$ isotope dilution method[11]. As the $^{15}N$ labeling of N components in the atmosphere is clearly impossible, the receiving pool (i.e., the soil/plant system; see Fig. 1) is labeled instead — an approach which is similar to the procedure used to determine the biological N fixation of legumes[12]. The airborne N input AN leads in the system S to the dilution of the $^{15}N$ tracer with the abundance used $a_T$ to produce the system abundance $a_S$. The $^{15}N$ balance in the system is described by the known isotope balance equation (Eq. 1):

$$n_T \times a'_T = (n_T + n_A + n_o) \times a'_S \quad (1)$$

$$n_T + n_A + n_o = n_S \quad (1.1)$$

$$AN_{gross} = n_A = n_T \times (a'_T/a'_S - 1) - n_o \quad (2)$$

$$AN_{net} = n_S\,'\,(1 - a'_S/a'_T) - n_o \quad (3)$$

where AN = atmospheric N deposition, a = $^{15}N$ abundance, a′ = $^{15}N$ excess, a′ = a =0.366 at.%; n = N amount in mg; A = atmosphere; S = system; T = tracer; and o = seed.

From this we can derive Eq. 2 for the gross airborne N input. Because there is always a loss of $^{15}N$ from the system (see “Results and Discussion”), the gross N input is not of practical relevance. Based on the total N actually contained in the system at the end of the measuring period ($n_S$) and referring to Eq. 1.1, one can deduce Eq. 3 for the net N input.

The principal set up of the ITNI system is shown in Fig. 2. Plants were cultivated on N-free quartz sand and supplied with a nutrient solution. A vegetation pot (1) according to Kick-Brauckmann with a surface of 0.038 m² was connected via a water drain to a collection vessel (2) for nutrient solution and rainwater surplus. From time to time, the rainwater and solution mixture was pumped by a peristaltic pump (5) onto the sandy surface of the pot. If the level of liquid dropped below a minimum, the vessels (2) were refilled with distilled water from a reserve vessel (3) via an automatic valve (4). To avoid anaerobic conditions inside the buffer vessel (2) which could cause N losses by denitrification, the vessel was aerated with clean air. The system contained four plant pots operating in parallel.

The nutrient solutions consisted of potassium, magnesium, and iron. Phosphorus in the form of $CaHPO_4$ was applied directly to the sandy surface. The $^{15}N$ tracer [$^{15}N$]ammonium-[$^{15}N$]nitrate ($^{15}NH_4{}^{15}NO_3$) was divided into 2–5 portions over the growing period of the crop and amounts totalling 200–600 mg N

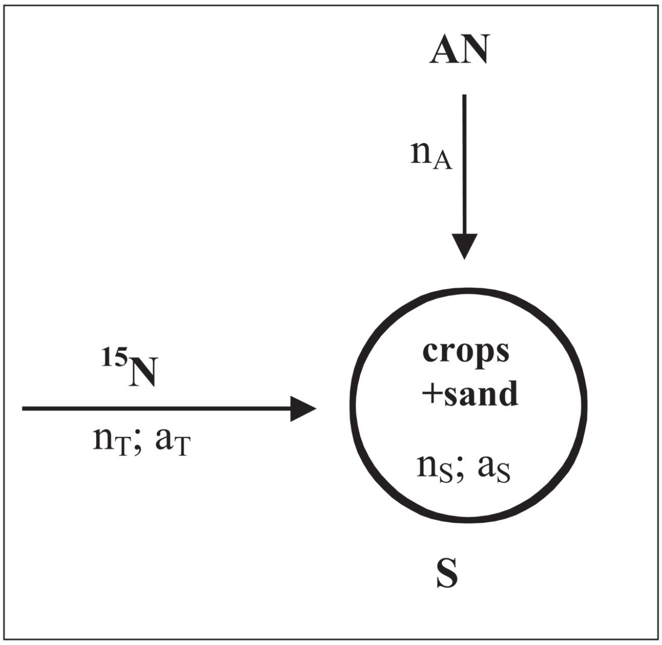

**FIGURE 1.** Model for the determination of airborne N input into a soil/plant system.

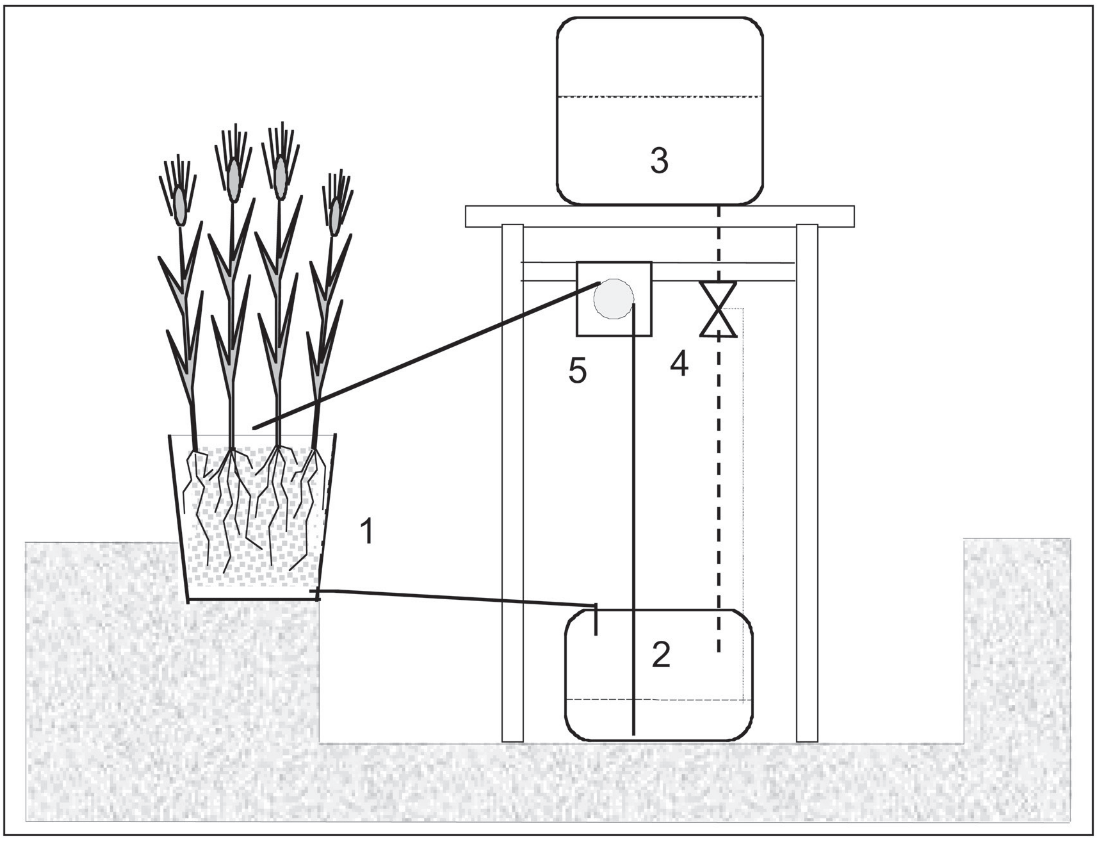

**FIGURE 2.** Diagram of the ITNI system: (1) vegetation pot, (2) buffer vessel for nutrient rainwater mixture, (3) vessel with distilled water, (4) electric valve, (5) peristaltic pump.

depending on the crop used. Suitable crops were various cereals and corn, although sunflower, rape, sugar beet, and green cabbage were also tested.

After harvest, the $^{15}N$ abundance in the plant, sand, and nutrient solution fractions was determined. The experimental procedure and $^{15}N$ analysis are described elsewhere in more detail[4,13].

## Study Sites

### *Bad Lauchstädt*

The field experimental station at Bad Lauchstädt is located about 20 km southwest of Halle/Saale. The area can be characterized as follows:

| | |
|---|---|
| Soil type: | Loess-chernozem (black earth soil) |
| Soil form (FAO): | Haplic Phaeozem |
| Altitude: | 113 m above sea level |
| Average annual precipitation (1996–1998): | 486 mm |
| Average temperature: | 8.7°C |

### *Etzdorf*

This site is also in the loess-chernozem region about 15 km northwest of Bad Lauchstädt. The ITNI system is located on farmland. Because of the rather low precipitation, this region is known as the dry belt of Central Germany.

## RESULTS AND DISCUSSION

The measurements directly obtained using the ITNI system for a vegetation period and their evaluation, as well as extrapolation to an area of 1 ha and the total for a year of observation, are shown in Table 1 by way of example for the measuring period 1997/1998. Only the net depositions according to Eq. 3 are included, as the gross values are irrelevant for practical purposes.

The relative methodological error, which is mainly caused by the complicated sample preparation process rather than the different analysis techniques used, was up to 7%. The variability between the four pots operated in parallel was much higher, being in the range of 5–25% for all the measurements carried out. As the variability between the pots far exceeds the methodological error, the former was listed in the tables as the degree of error.

$^{15}$N recovery was on average 80–90%. Values less than 80% also occurred, even though denitrification was supposed to have been minimized by the aeration of the buffer vessels. Plants are known to emit N in the form of ammonia into the atmosphere during the process of ripening (up to 5% of the plant N according to Schjoerring[14]). In order to eliminate these losses from the measurements, the corn was harvested some time before it was ripe. Nevertheless, there are also indications from $^{15}$N trials[15,16] that measurable quantities of assimilated $^{15}$N may even be emitted into the atmosphere in earlier vegetation stages. Such loss mechanisms could explain the sometimes low $^{15}$N recovery. However, as only the net N uptake calculated by Eq. 3 is used here, the $^{15}$N yield does not actually affect the results.

Extrapolation of the airborne N input from the vessel (Table 1, column 4) onto an area of 1 ha (Table 1, column 5) was performed based on the vessel surface area of 0.038 m$^2$.

The N deposited per year was calculated by adding the depositions calculated for the individual trial periods. Whenever the periods of two trials overlapped, a mean value was used. This procedure is shown as an example in Fig. 3.

As previously stated, in the current state-of-the-art, the atmospheric N deposition is only determined as inorganic N in the form of wet-only or bulk deposition. Consequently, the actual N input into the soil/plant system, which also includes gaseous deposition and N uptake by the aerial plant parts, is for methodological reasons bound to be underestimated.

Table 2 compares the wet-only and bulk depositions with the ITNI values based on the annual depositions from 1994–1995. The proportion of bulk deposition to the total N input is just 57%.

The total N inputs from the atmosphere calculated using the evaluation procedure described above are shown in Fig. 4 for Bad Lauchstädt, for which a complete series of measurements from 1994–2000 using the ITNI system exists. Leaving aside the value for 1997, which was not fully recorded by ITNI measurements, an average of 64 ± 11 kg N/ha × year results.

ITNI measurements at the second site in Central Germany, Etzdorf, only began in spring 1998. The annual depositions calculated so far at Etzdorf are as follows:

| | |
|---|---|
| 1998/1999: | 59.6 kg N/ha × year |
| 1999/2000: | 68.8 kg N/ha × year |
| Mean: | 64.8 kg N/ha × year |

**TABLE 1**
**Results of the ITNI Measuring Period 1997/98**

| Crop | Growing Period | Days | N Input mg N/pot | Deposition (g N/ha × d) |
|---|---|---|---|---|
| Winter rye | 09/97–04/98 | 221 | 90.6 ± 17.7 | 108 ± 21 |
| Spring barley | 03/98–06/98 | 87 | 80.9 ± 9.6 | 241 ± 27 |
| Corn | 05/98–07/98 | 84 | 120.8 ± 23.0 | 378 ± 72 |
| Green cabbage | 08/98–11/98 | 106 | 63.6 ± 5.1 | 158 ± 16 |

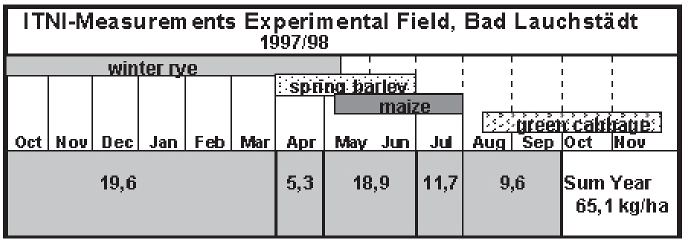

**FIGURE 3.** Calculation of the N deposition per year 1997/98 from the individual ITNI measurements.

**TABLE 2**
**Summarized Results of Different N Deposition Measurements from 1994/95 at Bad Lauchstädt**

| Type of Deposition | 1994 (kg N/ha × year) | 1995 (kg N/ha × year) | Mean 94/95 (kg N/ha × year) |
|---|---|---|---|
| Wet-only[a] | 12 | 11 | 11.5 |
| Bulk $N_t$ | 37 | 36 | 36.5 |
| ITNI | 62 ± 11 | 65 ± 4 | 63.5 ± 12 |
| Bulk/ITNI | 0.57 | 0.55 | 0.57 |

[a] Only ammonium-N + nitrate-N.

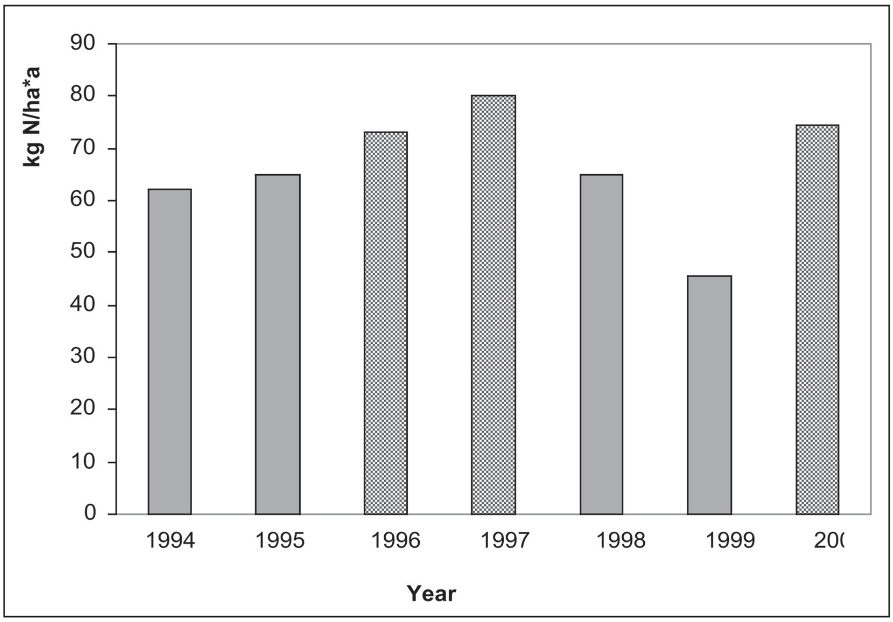

**FIGURE 4.** Total atmospheric N deposition from 1994–2000 at Bad Lauchstädt (1997 data incomplete).

The mean N deposition calculated for Etzdorf is equal to that of Bad Lauchstädt. However, owing to the high spatial and temporal variation, the N input for Etzdorf is not yet statistically confident.

The values calculated for Bad Lauchstädt agree well with the N deposition values derived from N balances at the long-term Static Fertilization Experiment in Bad Lauchstädt. For the period 1978–1996, they result in an average total atmospheric N input of 56.1 kg/ha[17]. These results indicate that an average N deposition of 60 ± 10 kg/ha × year must be expected on all agricultural areas in Central Germany. Similarly high values are confirmed by the results of other European long-term experiments such as in Askov (Denmark)[18], 46 kg/ha × year; Rothamsted (U.K.)[19,20], 48 kg/ha × year; and Prague (Czechoslovakia)[21], 61 kg/ha × year. Lower values were calculated for sandy sites such as Thyrow (Germany), 17 kg/ha × year, and Skierniewice (Poland), 31 kg/ha × year[22], probably because a considerable portion of N deposited was not taken up by plants but leached below the rooting zone. Other values on the same scale (some even exceeding 100 kg/ha × year) have been quoted for areas in the Netherlands and other European countries[23].

## CONCLUSIONS

Previously, the average atmospheric N deposition in Germany was estimated to be about 30 kg/ha × year. However, these values are based on measurements of wet-only and/or bulk depositions, and do not contain gaseous N deposition or direct N uptake by the plants.

The newly developed ITNI measuring system using $^{15}N$ enables the total atmospheric N input into a soil/plant system to be directly determined. It is based on the $^{15}N$ isotope dilution of a certain $^{15}N$ amount in a closed soil/plant system by the airborne N input (without taking into account biological $N_2$ fixation). At Bad Lauchstädt, a total average N deposition of 64 ± 11 kg/ha × year was calculated from 1994–2000. The indirect determination of the atmospheric N deposition from N balances of the Static Fertilization Experiment in Bad Lauchstädt gave values of 50–60 kg/ha ´ year. This order of magnitude is confirmed by other long-term trials in Germany and Europe.

The ITNI measurements and the values indirectly calculated from N balances reveal that the atmospheric N deposition was previously underestimated. N depositions on the scale determined exceed the critical loads for N of almost all close-to-nature ecosystems and hence constitute a considerable burden for them. This free N input for agriculture must be taken into account when calculating the amount of fertilizer to be applied. By including the total atmospheric N deposition into fertilization recommendations, an effective contribution can be made to reduce excess N. In the long term, this should also enable the reduction of N depositions, which is essential to protect close-to-nature ecosystems.

| | | |
|---|---|---|
| Received: | July | 13, 2001 |
| Revised: | September | 3, 2001 |
| Accepted: | September | 4, 2001 |
| Published: | October | 3, 2001 |
